# Transcatheter thrombolysis combined with damage control surgery for treatment of acute mesenteric venous thrombosis associated with bowel necrosis: a retrospective study

**DOI:** 10.1186/s13017-015-0045-2

**Published:** 2015-10-29

**Authors:** Kai Liu, Jiaxiang Meng, Shuofei Yang, Baochen Liu, Weiwei Ding, Xingjiang Wu, Jieshou Li

**Affiliations:** Medical School of Nanjing University, Nanjing, 210002 Jiangsu Province P. R. China; Research Institute of General Surgery, Jinling Hospital, 305 East Zhongshan Road, 210002 Nanjing, Jiangsu China

**Keywords:** Acute superior mesenteric venous thrombosis, Interventional thrombolysis, Damage control surgery, Bowel necrosis

## Abstract

**Objective:**

This study aims to evaluate the clinical outcomes of transcatheter thrombolysis in acute superior mesenteric venous thrombosis (ASMVT) associated with bowel necrosis.

**Methods:**

A retrospective study of six patients with ASMVT treated with catheter-directed thrombectomy/thrombolysis and damage control surgery at Jinling Hospital (Nanjing, China) between 2010 and 2013 was conducted. Demographics, past medical history, risk factors, therapeutic methods and effects, mortality, and follow-up of the study population were assessed.

**Results:**

Five of six patients underwent arteriovenous combined thrombolysis, while one patient underwent arterial thrombolysis. All patients required damage control surgery, and four of these patients underwent temporary abdominal closure. All patients survived and were free of recurrence.

**Conclusions:**

Transcatheter thrombectomy/thrombolysis and damage control surgery could help avoid extensive bowel resection, prevent short bowel syndrome and reduce mortality for critically ill patients with acute mesenteric venous thrombosis associated with bowel necrosis.

## Introduction

Acute superior mesenteric venous thrombosis (ASMVT) is an uncommon disease that presents no obvious specific symptoms in the early stage [1]. The diagnosis of ASMVT remains elusive until intestinal gangrene and peritonitis occurs [2]. The optimal time for treatment is lost, and eventually massive bowel resection is required, leading to short bowel syndrome [3–5]. Therefore, early diagnosis and prompt therapy is very important to improve the survival rate of patients with ASMVT, with the goal of conserving as much bowel as possible [6–8].

For patients with ASMVT associated with bowel ischemia, the recommended treatment is emergency surgery. However, in venous thrombosis, unlike arterial thrombosis, the line of demarcation is indistinct; leading to massive bowel resection. This is associated with the development of short bowel syndrome and high mortality rates [1, 9]. In order to improve the viability of the ischemic and congested bowel secondary to mesenteric venous thrombosis, a strategy of initial transcatheter thrombolysis followed by surgery has been adopted with success [10, 11].

In this study, we evaluated the outcome of this novel approach in critically ill patients combined with damage control surgery.

## Materials and methods

All procedures performed in studies involving human participants were in accordance with the ethical standards of the Ethics Committee of Jinling Hospital and with the 1964 Helsinki declaration and its later amendments or comparable ethical standards. Informed consent was obtained from all individual participants included in the study. Additional informed consent was obtained from all individual participants for whom identifying information is included in this article.

### Patient group

This is a retrospective study of patients with ASMVT treated between October 2010 and November 2013. Patients with ASMVT associated with bowel ischemia treated with transcatheter thrombolysis followed by damage control surgery were included in this study. Patient data were retrieved from medical records.

### Diagnosis and evaluation

All patients underwent routine blood examination, including: white blood cell, red blood cell, platelet and neutrophilic granulocyte count; hemoglobin, C-reactive protein, serum lactate and procalcitonin levels; and hepatic and renal function including amylase, lipase, aminotransferase, urea, creatinine and phosphocreatine kinase levels. Coagulation was monitored via thrombin time, activated partial thromboplastin time, international normalized ratio, fibrinogen, antithrombin III level, D-dimer and thromboelastogram every 24 h throughout the hospitalization. All patients underwent laboratory testing for thrombophilic states.

All patients underwent Doppler ultrasound scans and multislice spiral computed tomography (MSCT) to confirm ASMVT.

### Initial management

Each patient was treated with emergent treatment in the ICU to maintain hemodynamic stability. Vesical pressure was regularly monitored to observe intra-abdominal pressure changes. The use of vasopressors and somatostatin were avoided, because these could exacerbate intestinal ischemia. Systemic anticoagulation for superior mesenteric venous (SMV) thrombosis was carried out by immediate subcutaneous low molecular weight heparin injection. Dextran and alprostadil were used to improve microcirculation.

### Thrombolytic technique

Before catheter-directed thrombolytic therapy, the risk of bleeding complications and benefits of catheter-directed thrombectomy and thrombolysis for acute SMV thrombosis were explained to each patient, and informed written consent was obtained. Thrombolytic therapy was used for patients with persistent or worsening abdominal pain despite systemic anticoagulation.

Contraindications to percutaneous thrombolysis included previous stroke, central nervous system malignancies, active bleeding diathesis, and recent gastrointestinal bleeding. Direct portal vein (PV)-SMV thrombolysis was not performed in patients with unfavorable anatomy of the PV, marked atrophy of the liver, or portal venous cavernous transformation.

The route, thrombolytic agent, catheter type, length of treatment, other interventional techniques, and complications are shown in Table 1.

**Table 1:** Details of transcatheter thrombolysis therapy

No. of patients	location of thrombosis	Route of thrombolysis	Days of SMA thrombolysis	Agent and dose of SMA thrombolysis	Days of SMV thrombolysis	Agent and dose of SMV thrombolysis	Total dose of thrombolytics	Days of thrombolytic infusion
1	PV + SPV + SMV	SMA (first) + PT (next)	6	Urokinase (3,200,000 IU)	5	Urokinase (2,800,000 IU)	Urokinase (6,000,000 IU)	11
2	PV + SMV + IVC	SMA + PT	3	Urokinase (600,000 IU)	7	Urokinase (2,600,000 IU)	Urokinase (3,200,000 IU)	7
3	SMV	PT + SMA	6	urokinase (1,700,000 IU)	6	Urokinase (1,200,000 IU)	Urokinase (2,900,000 IU)	6
4	PV + SPV + SMV + IVC	SMA	5	Alteplase (60 mg) + urokinase (1,200,000 IU)	-	-	Alteplase (60 mg) + urokinase (1,200,000 IU)	5
5	PV + SMV + IVC	TI + SMA	3	Alteplase (10 mg) + urokinase (600,000 IU)	6	Alteplase (110 mg) + urokinase (400,000 IU)	Alteplase (120 mg) + urokinase (1,000,000 IU)	6
6	PV + SPV + SMV	PT + SMA	5	Alteplase (30 mg)	5	Alteplase (50 mg)	Alteplase (80 mg)	5

*SMV* superior mesenteric vein, *PV* portal vein, *SPV* splenic vein, *IVC* inferior vena cave, *PT* percutaneous transhepatic route, *TI* transjugular intrahepatic route, *SMA* superior mesenteric arterial route

### Direct PV-SMV catheterization

The choice between direct-catheter thrombolysis by percutaneous transhepatic or transjugular intrahepatic routes was determined by radiologists and the gastrointestinal surgeon based on the patient’s CT findings, vessel status and general condition.

The portal vein was punctured under ultrasonography and fluoroscopic guidance. After the injection of local anesthesia, catheterization of the right hepatic vein was performed using a 10-Fr, 41-cm long sheath (Rösch-Uchida TIPS Puncture Set; Cook, Bloomington, IN, USA) via the right transjugular approach. The catheter was advanced further to puncture the right portal vein. Once the portal puncture was successfully performed, an ultra-smooth guide wire (Terumo Europe, Leuven, Belgium) was introduced into the PV-SMV system. The SMV thrombus was traversed using a 5-Fr Cobra catheter (Cordis, Miami, USA). The thrombus in the PV-SMV system was aspirated as much as possible using an angled 8-Fr guiding catheter (Cordis, Miami, USA) with a 60-ml syringe through the 10-Fr sheath. After most of the thrombi in the PV-SMV were removed, a 5-Fr multiple side-hole catheter (Cordis, Miami, USA) was securely placed within the SMV thrombus.

Alternatively, the right portal vein was punctured directly with a 14-gauge trocar needle (Cook, USA) via the right midaxillary line under ultrasonography guidance. Then, a 6-Fr sheath (Cordis, Miami, USA) was introduced into the PV-SMV system. The rest of the procedure was the same as the prior TIPS method. After thrombolytic treatment, the punctured tract was sealed using a steel ring (Cook, USA) in order to prevent subcapsular hemorrhage.

### SMA Catheterization

The right femoral artery was cannulated using the Seldinger technique and a 5-Fr sheath (Cordis, Miami, USA) was placed. A 5-Fr Cobra catheter (Cordis, Miami, USA) was introduced; then, superior mesenteric arteriography and indirect portal and mesenteric vein venography were performed. Subsequently, selective catheterization of the superior mesenteric artery (SMA) was performed with a 5-Fr multiple side-hole catheter (Cordis, Miami, USA) in order to perform the indirect PV-SMV thrombolytic treatment.

### Transcatheter anticoagulant and thrombolytic therapy

Following the mechanical aspiration procedure, a bolus of 5 mg of rt-PA (alteplase; Boehringer Ingelheim GmbH, Germany) was injected via the catheter followed by continuous infusion of 0.625–0.833 mg/h for 24–48 h at the ICU (laboratory analysis and thromboelastogram were performed at 24-h intervals throughout the rt-PA infusion period). Then, rt-PA was replaced with urokinase (Nanjing Nanda Pharmaceutical Co. Ltd. Nanjing, China) at 400,000–600,000 IU/day for 4–6 days.

After completion of the thrombolytic therapy, the indwelling catheter was used to give low molecular heparin infusions at a rate of 417 IU/h; in order to maintain the activated partial thromboplastin time (APTT) at values two times the control value throughout the hospitalization.

### Termination of thrombolysis and timing of laparotomy

During transcatheter thrombolytic therapy, PV and SMV patency of patients were assessed every 24 h by ultrasonography. The angiogram was repeated via the infusion catheter at 24, 48 and 72 h and one week following the procedure. Contrast-enhanced CT scan was performed upon completion of thrombolytic therapy or in patients with signs of peritonism or bloody ascites.

Termination of thrombolysis was based on clinical and radiographic findings. Particularly, when CT demonstrated intestinal perforation, transmural bowel necrosis, and abdominal paracentesis revealed bloody ascites. Patients with these findings were treated by laparotomy as soon as possible. Some abnormal serum markers and intra-abdominal pressure are shown in Table 2. During surgery, resection of the gangrenous bowel with double barrel enterostomy was performed. In the presence of intra-abdominal hypertension, the abdomen was left open. Abdominal closure was performed once intra-abdominal pressure returned to normal range. Definite intestinal anastomosis was performed after patients completely recovered from the acute phase and were on a full oral diet (Table 3).

**Table 2:** Biochemical and hematological parameters of patients 24 h before exploratory laparotomy

No. of patients	WBC (10^9^/L)	CRP (mg/L)	D-dimer (mg/L)	PCT (μg/L)	Albumin (g/L)	Lactate (mmol/L)	LDH (U/L)	LAP (U/L)	IAP (cmH_2_O)
1	19	144	2.55	21.16	34.3	19.6	230	61	8
2	19.4	140.6	2.25	23.94	29.4	17.6	171	65	10
3	39.1	190	1.45	12.26	30.5	21.3	339	76	13
4	19.9	189	18.79	55.27	27.9	16.4	437	78	5
5	32.9	150	8.55	5.13	30.0	25.8	980	65	9
6	22.8	151.3	3.54	14.45	24.5	15	959	103.9	6

Reference range: white blood cell (WBC): 3.5–9.5 × 10^9^/L, Creactive protein (CRP): 0–8 mg/L, D-dimer: 0–0.55 mg/L, procalcitonin (PCT): 0–0.5 μg/L, Albumin: 40.0–55.0 g/L, Lactate: 0.5–1.6 mmol/L, lactate dehydrogenase (LDH): 109–245 U/L, leukocyte alkaline phosphatase (LAP): 30–120 U/L, intra-abdominal pressure (IAP): 0 kPa. 1 kPa = 10.2 cmH_2_O

**Table 3:** Perioperative and follow up data of patients

No. of patients	Days from thrombolysis to surgery	Initial findings and treatment	Range of small bowel resection	Days of open abdomen (temporary abdominal closure)	Days of hospital stay	Timing of definitive surgery (months)	Findings and definitive surgery	Months of follow-up	Clinical outcome
1	7	Bowel resection, double barrel enterostomy and open abdomen^a^	200 cm	3	27	12	No further intestinal necrosis.	37	No recurrence
							double-cavity stoma reversion		
2	7	Bowel resection, double barrel enterostomy and open abdomen^a^	200 cm	3	47	12	No further intestinal necrosis.	32	No recurrence
							double-cavity stoma reversion		
3	7	Bowel resection, double barrel enterostomy and open abdomen^a^	150 cm	7	29	12	No further intestinal necrosis.	25	No recurrence
							double-cavity stoma reversion		
4	18	Bowel resection and double barrel enterostomy	150 cm	-	70	12	No further intestinal necrosis.	20	No recurrence
							double-cavity stoma reversion		
5	8	Bowel resection, double barrel enterostomy and open abdomen^a^	250 cm	3	38	12	No further intestinal necrosis.	16	No recurrence
							double-cavity stoma reversion		
6	6	Bowel resection and double barrel enterostomy	80 cm	-	40	13	No further intestinal necrosis.	11	No recurrence
							double-cavity stoma reversion		

^a^Open abdomen was treated with temporary abdominal closure using Prolene Mesh

### Follow-up

After recovery from transcatheter thrombolysis and damage control surgery, patients were discharged on oral anticoagulation, which was adjusted to maintain prothrombin time at 4–6 s higher than normal values for six months.

Contrast enhanced CT and ultrasonography of the portal vein and SMV was conducted at discharge, followed by repeat imaging every three months for the first year, and every 6–12 months thereafter. Records of hospitalization and clinic visits were reviewed from the time of discharge until October 31, 2014.

## Results

### Patient demographics

Among these six patients, three were male and three were female; and mean age of patients was 41 ± 14.38 years (range, 20–59 years). Initial symptom of all patients was abdominal pain, which was accompanied by the following symptoms: abdominal distension (*n* = 5), nausea and vomiting (*n* = 3), and hematemesis (*n* = 1). All six patients experienced abdominal pain for less than two weeks (range, 5–14 days; mean, 9 ± 3.82 days). Related past medical history and risk factors in our study are presented in Table 4.

**Table 4:** Summary of clinical data

No. of patients	Age (years)/ gender	Initial symptoms and signs	Etiologies	Past medical history and risk factors	Indication for intervention	Days from symptoms to admission	Days from symptoms to intervention
1	46/M	Abdominal pain, distention	Portal hypertension	Portal hypertension	Continued pain, despite anticoagulation	5	7
2	51/M	Abdominal pain, distention	Pancreatitis and hypercoagulable state	Pancreatitis, deep venous thrombosis	Progressive pain, despite anticoagulation	14	17
3	29/F	Abdominal pain, distention	Post partum, IgA nephropathyand hypercoagulable state	Post partum, IgA nephropathy, deep venous thrombosis	Worsening pain, despite anticoagulation	7	7
4	41/F	Abdominal pain, nausea, anorexy	Hypercoagulable state with oral contraceptive agent	Oral contraceptive agent	Worsening pain, despite anticoagulation	8	9
5	20/M	Abdominal pain, distention, vomiting, diarrhea, hematemesis	Hypercoagulable state with activated protein C deficiency	Deep venous thrombosis, inferior vena cava thrombosis, pulmonary embolism	Persistent pain, distension, bloody ascites, despite anticoagulation	7	8
6	59/F	Abdominal pain, distention, vomiting, nausea,	Myeloproliferative disorders	None	Worsening pain, distension, despite anticoagulation	7	11

**Fig. 1:**
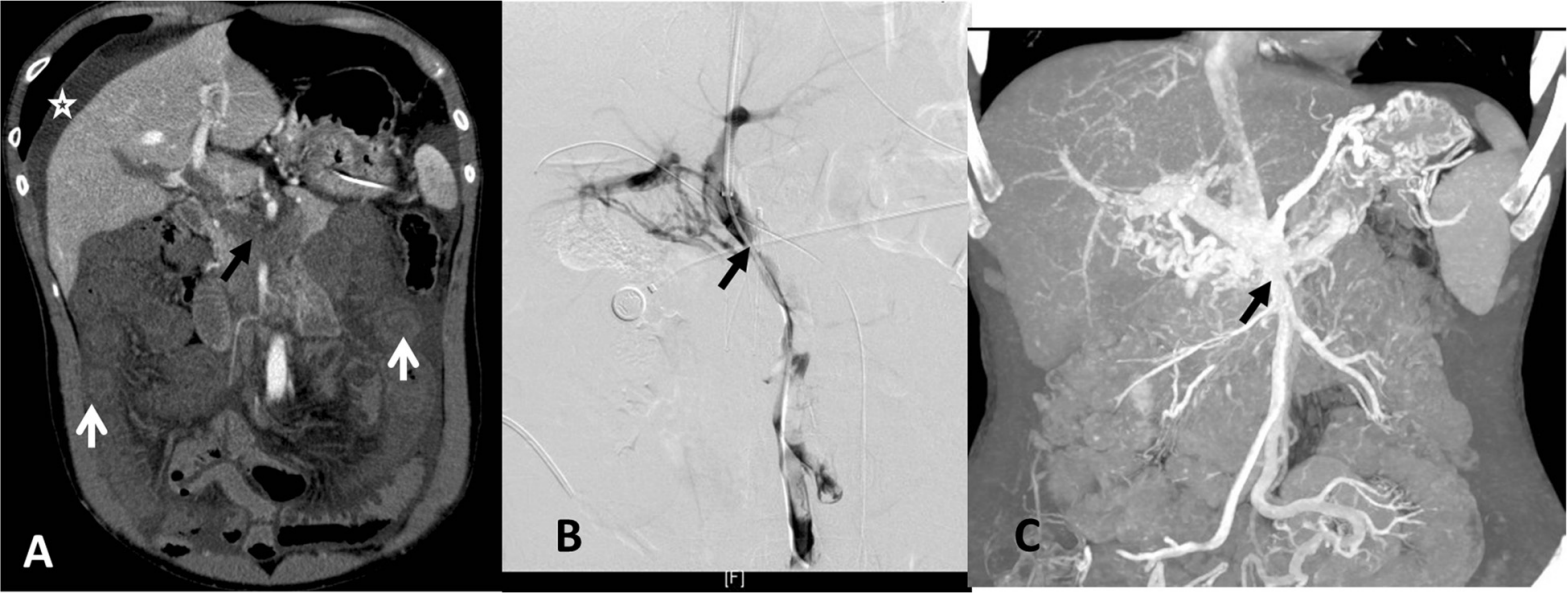
Patient No. 5: **a** MSCT demonstrates the massive thrombosis of the portal vein (PV) and superior mesenteric vein (SMV) (black arrow), diffuse circumferential bowel wall thickening with “halo sign” (white arrows), and the small amount of ascites (star). **b** Venography of the superior mesenteric and portal veins demonstrates filling defects in the lumen of PV and SMV via the percutaneous transhepatic route (black arrow). **c** MSCT demonstrates partial recanalization of PV and SMV (black arrow) and collateral vessels 12 months after surgery

**Fig. 2:**
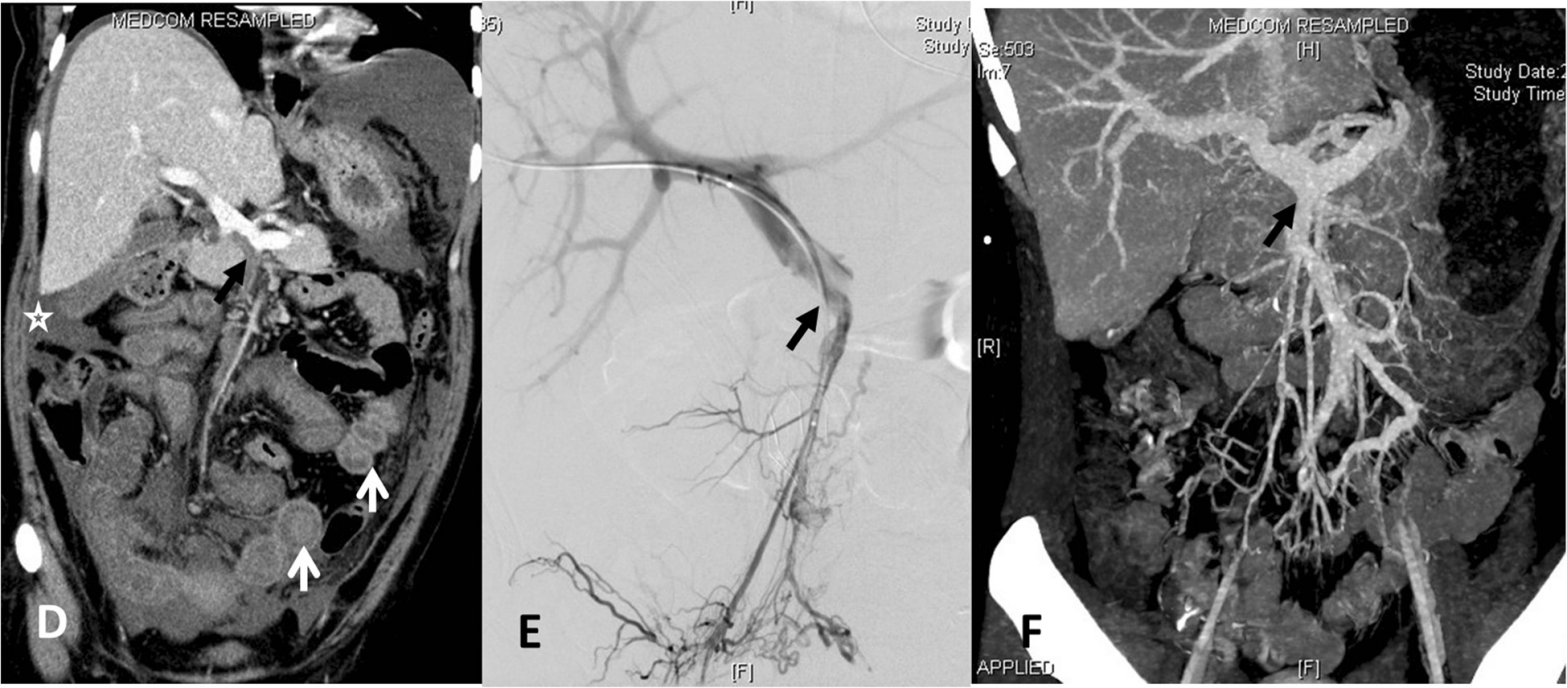
Patient No. 3: **d** MSCT demonstrates the massive thrombosis of the portal vein (PV) and superior mesenteric vein (SMV) (black arrow), diffuse circumferential bowel wall thickening with “halo sign” (white arrows), and the small amount of ascites (star). **e** Venography of the superior mesenteric and portal veins demonstrates filling defects in the lumen of PV and SMV via the transjugular intrahepatic route (black arrow). **f** MSCT demonstrates partial recanalization of PV and SMV (black arrow) at discharge

ASMVT was diagnosed and evaluated by MSCT. All patients underwent MSCT to detect SMV thrombosis and bowel affected by ischemia (Figs. 1 and 2). Based on clinical symptoms and CT findings, all patients underwent thrombolytic therapy.

### Thrombolytic therapy

Six patients were successfully treated with direct PV-SMV thrombolysis and SMA thrombolysis (Figs. 1 and 2). Patient No. 1 first received trans-SMA thrombolysis for six days, and was treated with trans-SMV thrombolysis for 5 days. Patient No. 4 only received SMA thrombolysis therapy for 5 days due to failure of direct PV-SMV catheterization. Catheter-directed mechanical thrombectomy was performed in three patients. Continuous thrombolysis via the infusion catheter in the SMV was provided for 5–7 days (average, 5.8 ± 0.84 days). The mean doses of thrombolytics via SMV access were: alteplase, 80 ± 42.43 mg; urokinase, 1.75 ± 1.15,000 000 IU. SMA trans-catheter thrombolysis lasted for 3–6 days (average, 4.67 ± 1.37 days). The mean doses of thrombolytics via SMA access were: alteplase, 33.3 ± 25.12 mg; urokinase, 1.46 ± 1.08,000 000 IU. The total doses of thrombolytics were: alteplase, 86.67 ± 30.55 mg; urokinase, 2.86 ± 2.01,000 000 IU. After completion of the infusion of thrombolytic drugs, venography via the infusion catheter was performed to observe the thrombolytic effect on SMV thrombosis. MSCT images were obtained again before surgery, which revealed the recanalization of SMV and the necrotic intestine.

### Damage control surgery

**Fig. 3:**
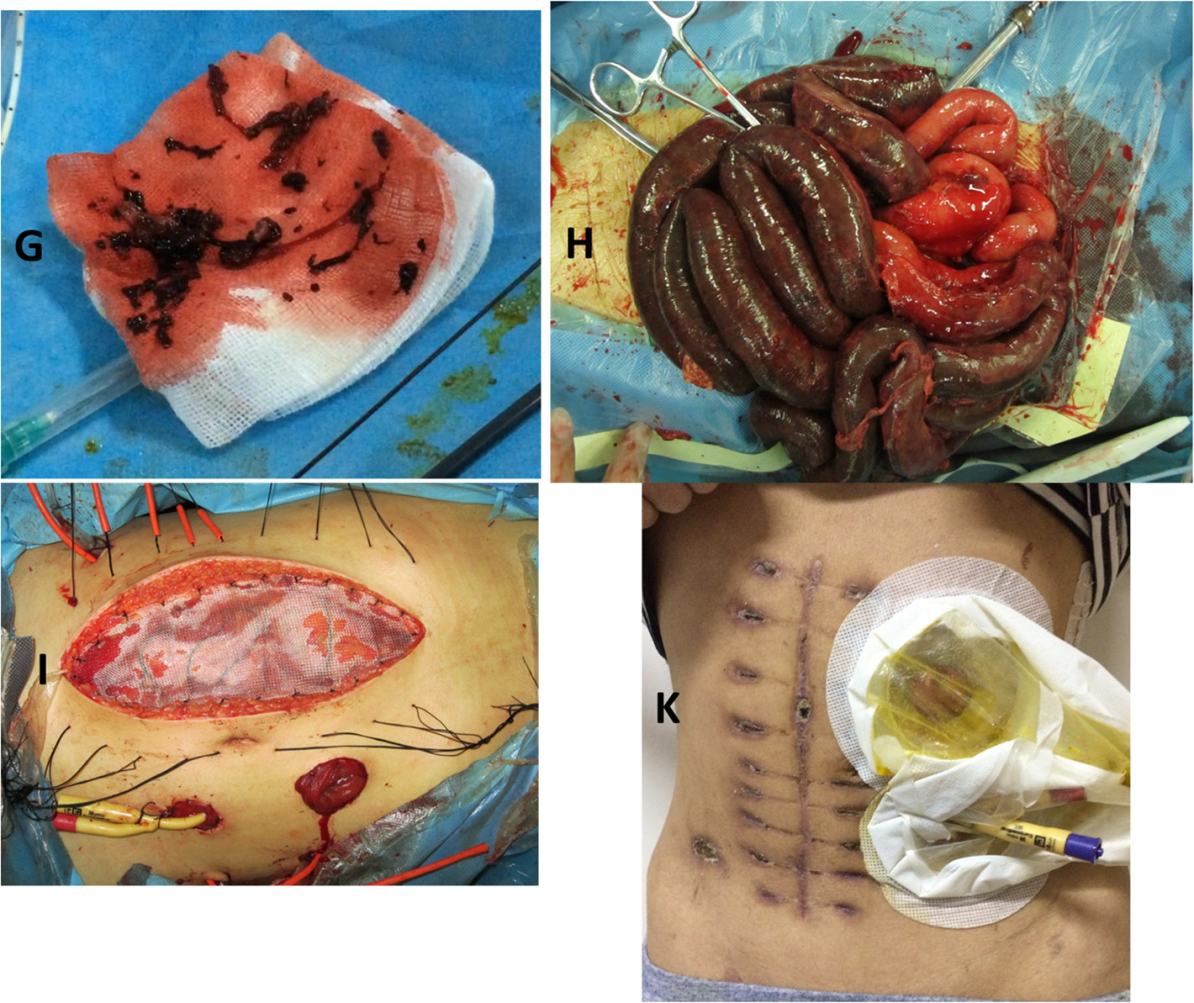
Patient No. 5: Venous thromboembolus (**g**) aspirated from the superior mesenteric vein via the catheter. Damage control surgery after thrombolysis: obvious transmural necrosis of the small intestine (**h**) temporary abdominal closure, and double stoma (**i**). Abdominal wound (**k**) at discharge after damage control surgery is shown

All six patients underwent exploratory laparotomy due to worsening of symptoms with the development of hemodynamic instability. During the procedure, hemorrhagic ascites were found along with intestinal gangrene and mesenteric edema. SMA pulsations were palpable, but mesenteric veins of the bowel were dilated and filled with red thrombi. Necrotic bowel resection and double barrel enterostomy were performed in all patients, in which four patients also underwent open abdomen due to sustained intra-abdominal hypertension. Mean length of the resected bowel was 171.67 ± 58.45 cm (range, 80–250 cm; Fig. 3)

Definitive surgery for restoring intestinal continuity was performed 6–12 months later.

### Complications

During thrombolytic infusion via the indwelling catheter, a small hematoma was observed in three patients at both the arterial or venous puncture site; which were treated conservatively without stopping thrombolytic therapy. Sepsis occurred in one patient, while intestinal perforation occurred in another patient before open exploration.

### Follow-up

None of the patients required long-term parenteral nutrition. In addition, there was no mortality or thrombosis recurrence until the last follow up. Mean duration of hospitalization was 41.83 ± 15.63 days (range, 27–70 days), and mean duration of follow-up after discharge was 23.5 ± 9.81 months (range, 11–37 months).

## Discussions

ASMVT is a rare but potentially catastrophic clinical complication that may lead to ischemia and/or infarction of the intestine [1, 4, 5]. The mortality rate of acute mesenteric venous thrombosis is usually up to 50 % [9]. The exact pathophysiology of acute SMV thrombosis remains unclear. However, it has been associated with some risk factors for thrombophilia such as myeloproliferative disease [12], coagulation factor deficiency, factor V Leiden [13] and prothrombin gene mutation [14], portal hypertension, post-splenectomy, malignancy, intra-abdominal infection or inflammation [15], pregnancy, oral contraceptive use, and so on.

Transcatheter thrombolysis is used to treat patients with ASMVT and bowel ischemia to achieve early recognition and mesenteric recanalization, and subsequently salvage the adjacent bowel with reversible ischemia. Operative intervention is reserved for patients with severe and diffuse peritonitis, and transmural bowel infarction [16]. Damage control surgery is performed to remove the gangrenous bowel with minimum surgical stress, in order to accelerate recovery from acute illness before performing definitive surgery [17, 18].

In the absence of clear indications for surgery, systemic anticoagulation therapy should be started as soon as possible [6, 8]. Interventional catheter thrombolysis may be more effective than systemic anticoagulation. For acute thrombosis, local thrombolytic therapy via a catheter introduced into the superior mesenteric vein or artery has all been shown to have gratifying results [19–23]. The indirect and continuous infusion of fibrinolytic therapy through the SMA is effective, because it enables the thrombolytic agent to disperse from the capillaries and small venules, tertiary and secondary arcades before reaching the larger veins [22, 23]. Transarterial infusion can reduce the side effects of drugs such as bleeding, and avoid intraperitoneal and hepatic subcapsular bleedings that may result from the association of liver puncture and the use of fibrinolytic agents and heparin [24]. Direct access to the portal system targets SMV thrombosis by a transjugular or transhepatic route, leading to rapid thrombus removal and venous flow re-establishment, and an improvement in symptoms [20–22]. Compared to the indirect method, direct thrombolysis is more efficient, less time-consuming, and provides the opportunity to perform mechanical aspiration and balloon angioplasty with possible stent placement for elastic recoil or persistent stenosis.

We conducted arteriovenous combined thrombolysis for these patients to reduce the need for greater lytic agent doses and longer durations of infusion therapy [22, 23]. There is no consensus on the best fibrinolytic agent to be used and its correct dosage [25]. In our study, alteplase was first used with a significantly smaller initial bolus (5 mg), and was maintained for 24–48 h at the recommended safe 0.625–0.833 mg/h infusion rate. It has been reported that t-PA has demonstrated a higher thrombolytic rate, less plasminogen activation, less α2-antiplasmin consumption and less fibrinogen breakdown when compared to urokinase [25]. Consequently, when the effect of urokinase was not obvious, we switched and used alteplase (tissue-type plasminogen activator), which can selectively dissolve blood clots. However, we cautiously reduced the dose of t-PA when the risk of bleeding was taken into account.

Contrary to previous thinking, surgical exploration is not necessary in all patients with mesenteric venous thrombosis. Premature laparotomy would lead to extensive resection of the small intestine, increase post-operative mortality, and affect the clinical outcome of patients [10, 16].

**Fig. 4:**
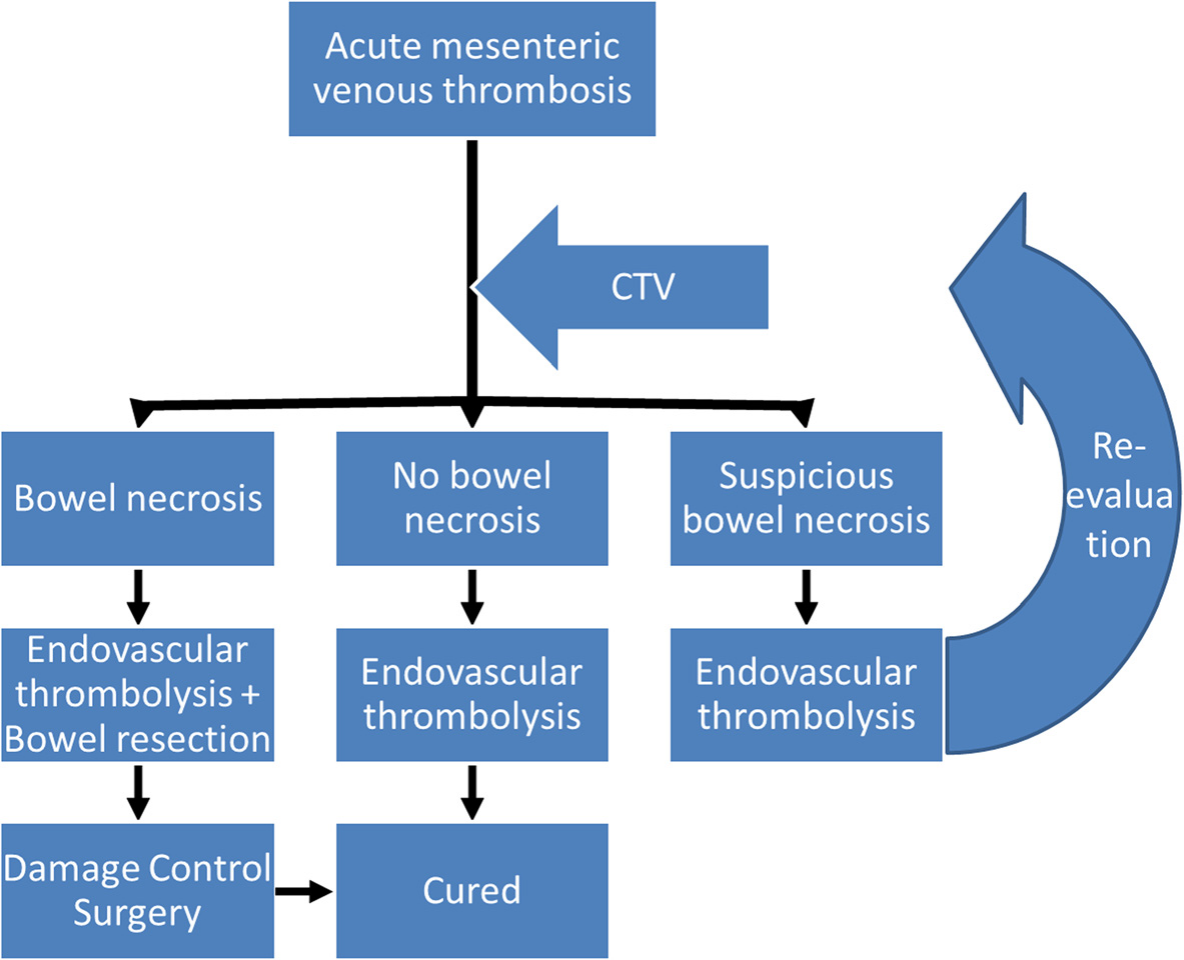
Proposed treatment strategy for acute mesenteric venous thrombosis is shown

From our own perspective (Fig. 4), the first step is to evaluate the extent and severity of intestinal ischemia. Unfortunately, there are no precise markers that can identify patients who are at risk for bowel infarction [2]. CT and laparoscopy may play a role in the diagnosis of transmural bowel infarction [7, 26]. Prolonged stasis of venous flow can cause arterial spasm, aggravating intestinal ischemia. Therefore, in patients with suspected acute mesenteric ischemia, revascularization is preferentially started prior to bowel surgery [16]; and anticoagulation treatment should be started immediately [6]. Compared to arterial thrombosis, intestinal ischemia caused by venous thrombosis has a slow development. It is noteworthy to mention that ischemic lesions occur first in the mucosa, which may be extensive. Thrombolytic therapy should be performed angiographically in an attempt to avoid further infarction. Previous studies have emphasized that longer durations of ischemia prior to treatment are associated with poorer prognosis [27]. Early catheter thrombolysis can restore blood flow to ischemic bowel and achieve maximum retention of the remaining intestine.

If bowel ischemia fails to reverse, intestinal gangrene occurs; leading to sepsis. In such cases, damage control surgery like bowel resection with double barrel enterostomy is preferred over complex definitive procedures in order to reduce second hit phenomenon in such critically ill patients [11, 17].

Double barrel enterostomy allows the observation of the color and vitality of the stoma, which provides an indirect assessment of the intestinal blood supply and avoids a second-look operation. Sometimes, temporary abdominal closure with prolene Mesh (Ethicon, LLC, USA) may be applied when there is severe bowel edema with risk of intra-abdominal hypertension or abdominal compartment syndrome [18].

This study has some limitations. Firstly, this is a retrospective study with a small sample size; hence, further comparative prospective trials are required to validate the findings of this study. Secondly, transcatheter thrombolysis can cause delay in damage control surgery, which can lead to bowel perforation, sepsis and deterioration of the patient’s clinical condition; as seen in one of our patients. Hence, continuous close monitoring is required during thrombolytic therapy in order to prevent undue delay in surgery.

## Conclusions

For critically ill patients with acute mesenteric venous thrombosis, transcatheter thrombectomy and thrombolysis may be useful to restore blood flow, improve intestinal ischemia, and avoid extensive bowel necrosis. By using the damage control strategy, necrotic bowel is resected, metabolic disturbance is corrected, and finally, definitive surgery is completed. The stepwise surgical approach for ASMVT needs further researches to provide more evidence-based data, to improve survival and prognosis of patients [11].
